# Bilateral extraocular muscle enlargement and proptosis associated with squamous cell carcinoma of the lung

**DOI:** 10.1259/bjrcr.20180049

**Published:** 2018-09-01

**Authors:** David I Kim, Gregory Lock

**Affiliations:** 1 Department of Diagnostic Radiology, Princess Alexandra Hospital, Woolloongabba, QLD, Australia

## Abstract

Thyroid-associated orbitopathy is characterised on cross-sectional imaging by symmetric extraocular muscle enlargement sparing the musculotendinous junction. We report a case of this imaging finding in a biochemically euthyroid patient with metastatic squamous cell carcinoma of the lung undiagnosed at time of presentation.

## Clinical presentation

A 63-year-old male presented to the emergency department with an acute episode of blurry vision and two months of headache and intermittent nausea. The patient denied any pain or infectious symptoms. Eye examination revealed bilateral proptosis with lid retraction, ophthalmoplegia in all directions, reduced visual acuity but no visual field defects. Physical examination was otherwise unremarkable.

## Investigations

**Figure 1.**
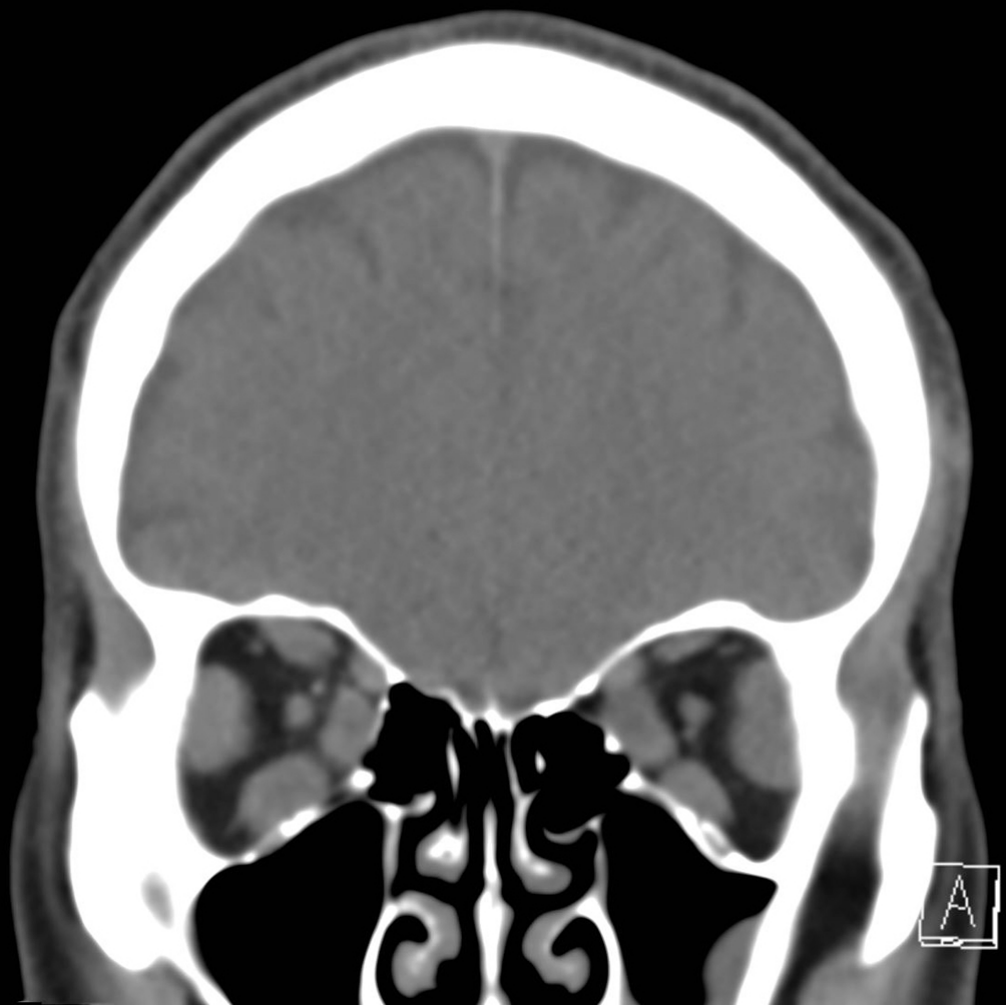
Coronal non-enhanced CT of the head demonstrates symmetric enlargement of the extraocular muscles in a 63-year-old male presenting with an acute episode of blurry vision and two months of headache and intermittent nausea.

A non-enhanced CT of the head was performed to further evaluate. This revealed significant enlargement of all extraocular muscles involving the left and right sides symmetrically (Figure 1) with sparing of the musculotendinous junctions, suggestive of thyroid-associated orbitopathy.

Subsequent thyroid function tests, however, were within normal limits. Anti-thyroid peroxidase and anti-thyroglobulin antibodies were negative.

**Figure 2.**
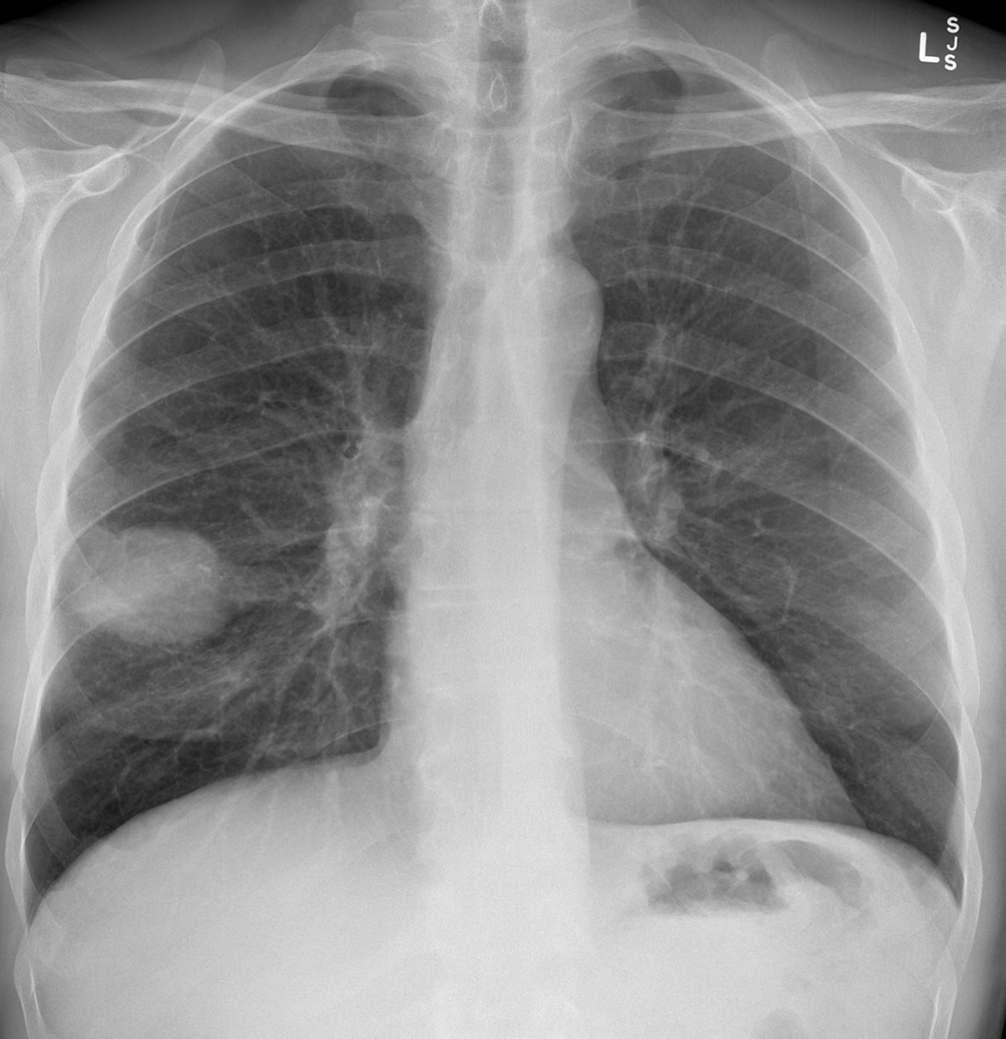
A large right middle lobe mass is identified on screening chest radiograph. On revisitation of history, 22 kg of unintentional weight loss over 3 months and a 40 pack-year smoking history were revealed.

On revisitation of the patient’s history, 22 kg of unintentional weight loss over the preceding 3 months and a 40 pack-year smoking history were elicited. A screening chest radiograph revealed a large right middle lobe mass (Figure 2).

**Figure 3.**
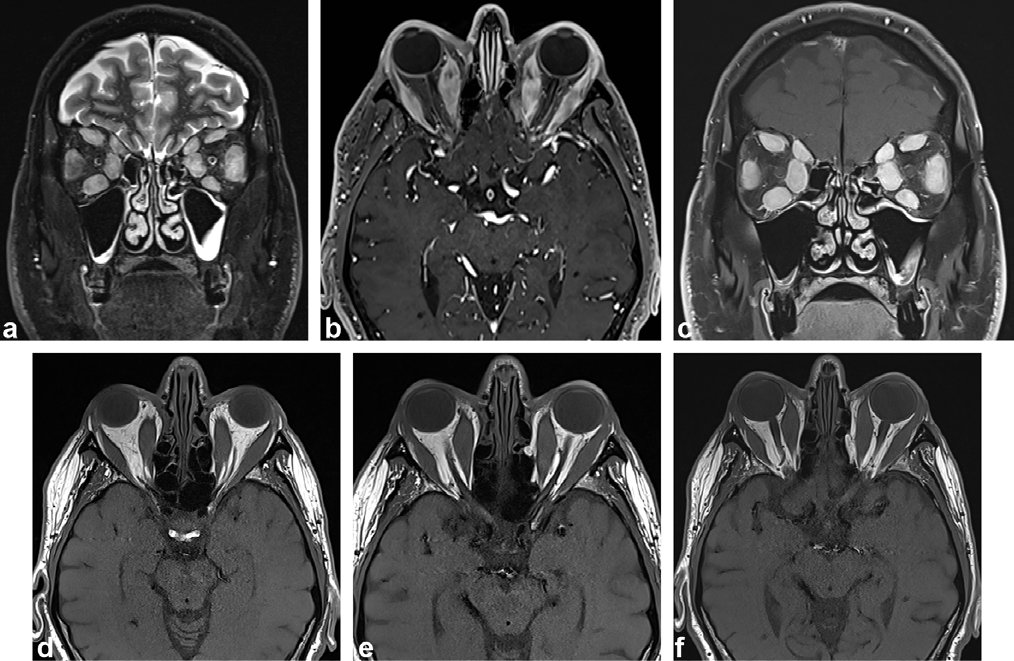
(a–f) Contrast-enhanced MRI of the orbits demonstrates symmetric enlargement of the extraocular muscles. Coronal STIR and axial and coronal contrast-enhanced fat-suppressed *T*_1_-weighted images (a–c) show increased signal within the enlarged, enhancing muscles with intra and extraconal oedema. Sequential axial *T*_1_-weighted images (d–f) illustrate sparing of the musculotendinous junctions. STIR, short tau inversion recovery.

No alternative or additional findings for the patient’s orbitopathy were demonstrated on a contrast-enhanced MRI of the head and orbits (Figure 3). Serum vasculitic screen, serum angiotensin converting enzyme levels, parathyroid hormone studies and urinary paraprotein studies were unremarkable. Serum potassium and corrected calcium were borderline elevated at 5.5 and 2.63 mmol.L^−1^ respectively but all other serum electrolytes, including ionised calcium, were within normal limits. Paraneoplastic autoantibodies, including anti-Hu, anti-Ri, anti-Yo, anti-PCA-2, anti-VGCC and anti-VGKC, were negative.

Contrast-enhanced CT of the chest, abdomen and pelvis revealed a solitary right middle lobe mass with ipsilateral and contralateral enlarged mediastinal lymph nodes. An endobronchial ultrasound-guided biopsy of an enlarged subcarinal lymph node and right middle lobe brushings and washings were subsequently performed; histopathological findings were consistent with primary lung squamous cell carcinoma of the right middle lobe with mediastinal nodal metastatic disease.

## Differential diagnosis

The differential for bilateral symmetric extraocular muscle enlargement can initially be narrowed by evaluating for involvement of the musculotendinous junction, with sparing suggestive of thyroid-associated orbitopathy. Given the distribution of involvement and absence of an infiltrative process causing this enlargement on MRI, other differentials such as lymphoma, metastasis, pseudotumour, sarcoidosis and amyloidosis were considered less likely.

## Treatment

**Figure 4.**
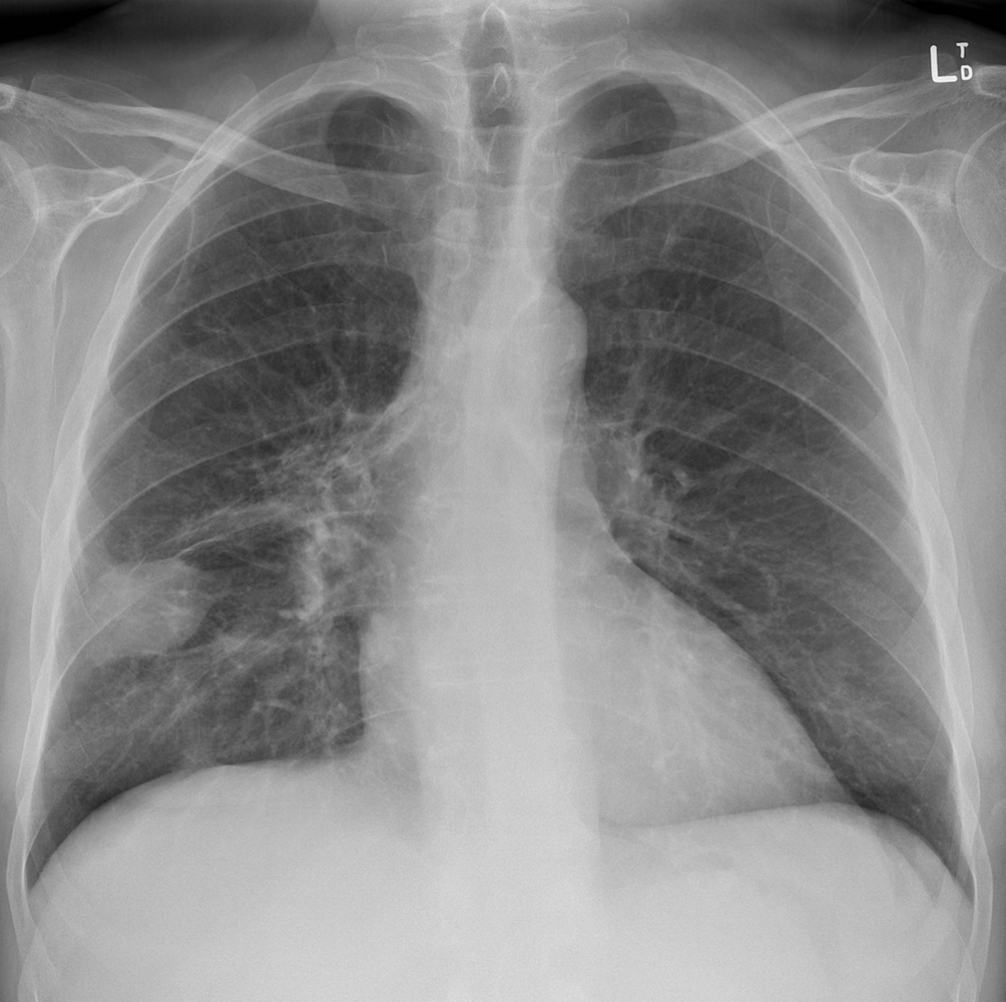
The right middle lobe mass, first imaged 4 months prior, has reduced in size after administration of chemoradiotherapy following a histopathological diagnosis of primary lung squamous cell carcinoma.

**Figure 5.**
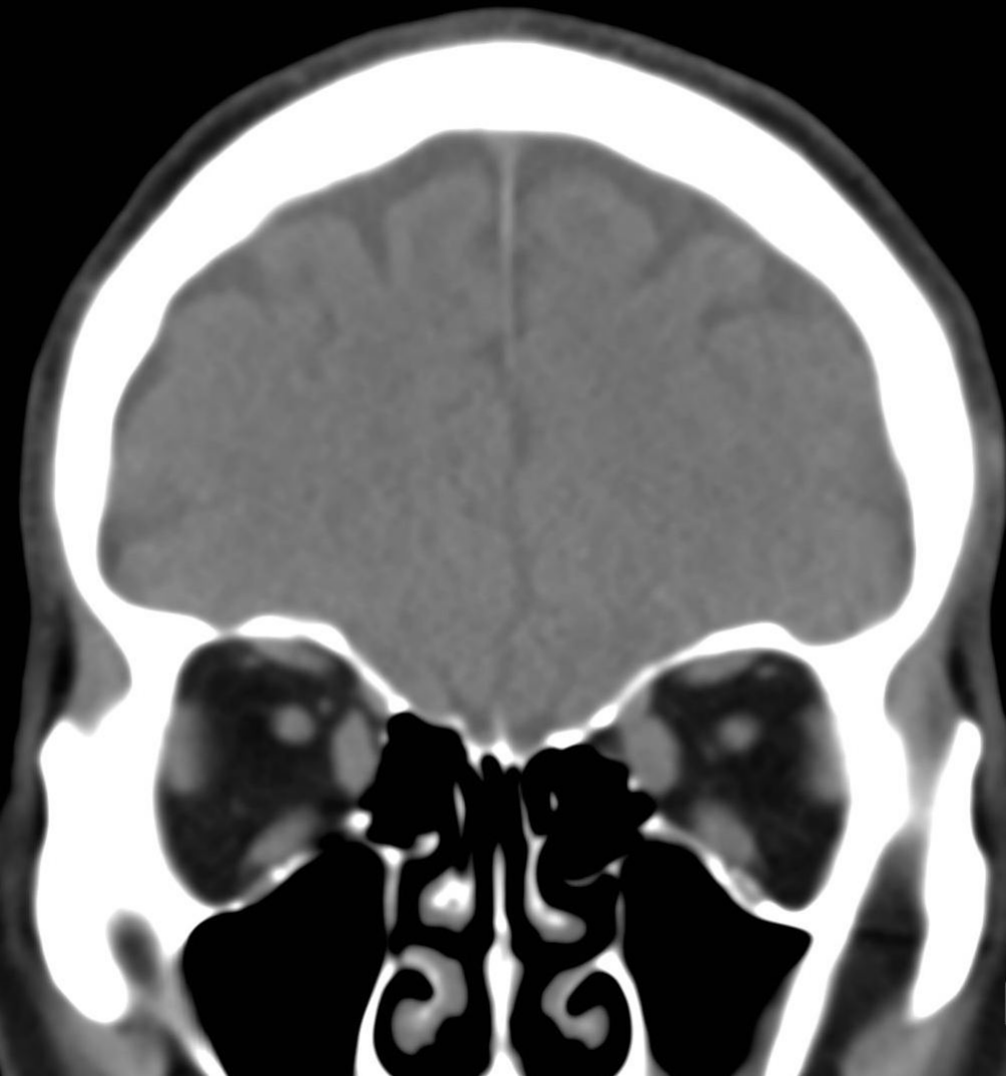
Coronal non-enhanced CT of the head demonstrates near complete resolution of bilateral extraocular muscle enlargement after completion of treatment for primary lung squamous cell carcinoma.

A treatment plan with curative intent comprised of 60 Gy in 30 fractions of radiotherapy and concurrent carboplatin and paclitaxel was prescribed. Ophthalmoplegia and visual symptoms improved with lubricating eye drops and 2 mg of oral dexamethasone daily. Post-treatment imaging was performed approximately 4 months after initial presentation. Chest radiograph revealed reduction in size of the primary right middle lobe malignancy with surrounding likely radiotherapy-related changes (Figure 4). Contrast-enhanced CT of the head, chest, abdomen and pelvis confirmed this radiographic finding as well as minimally reduced mediastinal lymphadenopathy, the absence of new metastatic disease and near complete resolution of the extraocular muscle enlargement (Figure 5).

## Discussion

Symmetric enlargement of all the extraocular muscles with sparing of the myotendinous junctions is a typical feature of Graves disease.^1^ Although there are published case reports of exophthalmos associated with primary lung malignancy, none to date have been associated with the CT and MRI orbital findings demonstrated in the current case.^2,3^ Concurrent response of the extraocular muscle enlargement to chemoradiotherapy for the right middle lobe primary malignancy on follow-up imaging reaffirms this association.

Lymphoma and metastasis have been reported to involve a single muscle, pseudotumours usually cause enlargement of the muscle and tendinous insertion, and MRI in sarcoidosis typically demonstrates enlarged, enhancing lacrimal glands.^4–6^ A German language article reported a case of enlargement of all the extraocular muscles associated with known hormonally inactive paraganglioma and suggested a paraneoplastic immune reaction as the cause.^7^ Unfortunately, the patient refused any treatment, precluding assessment as to whether orbitopathy would improve with treatment of the paraganglioma, and died 18 months later.

When imaging is consistent with thyroid-associated orbitopathy, and clinical and biochemical findings are incongruent, we suggest an occult malignancy should be sought. Contrast-enhanced MRI can define the structures involved. Paraneoplastic autoantibodies are variably helpful, given they are specific for paraneoplastic neurological disorders, particularly in the setting of any lung malignancy other than small cell carcinoma.^8^ Imaging should be targeted based on clinical suspicion; a heavy smoking history prompted a screening chest radiograph in the current case, which proved critical in this patient’s care.

## Learning points

1. This is the first reported case of subsequently discovered primary lung squamous cell carcinoma presenting with imaging proven bilateral symmetric extraocular muscle enlargement typical of Graves disease in the absence of clinical and biochemical evidence of Graves disease.
2. Enlargement of all extraocular muscles sparing the musculotendinous junctions and involving the left and right orbits symmetrically is typically associated with Graves disease.
3. Thyroid-associated orbitopathy in the absence of thyroid disease and presence of malignancy is rare but has been previously reported.
4. If imaging findings are entirely typical of thyroid-associated orbitopathy and clinical and biochemical assessment is incongruent, imaging, preferably targeted, should be performed with the aim of revealing an occult malignancy.
